# Oncolytic Viruses in the Era of Omics, Computational Technologies, and Modeling: Thesis, Antithesis, and Synthesis

**DOI:** 10.3390/ijms242417378

**Published:** 2023-12-12

**Authors:** Laura Menotti, Andrea Vannini

**Affiliations:** Department of Pharmacy and Biotechnology, University of Bologna, 40126 Bologna, Italy; andrea.vannini5@unibo.it

**Keywords:** oncolytic virus, oncolytic immunovirotherapy, cancer therapy, OV combination therapy, immunopeptidomics, cancer theranostics, virus synthetic biology, infection mathematical modeling, infection computational modeling

## Abstract

Oncolytic viruses (OVs) are the frontier therapy for refractory cancers, especially in integration with immunomodulation strategies. In cancer immunovirotherapy, the many available “omics” and systems biology technologies generate at a fast pace a challenging huge amount of data, where apparently clashing information mirrors the complexity of individual clinical situations and OV used. In this review, we present and discuss how currently big data analysis, on one hand and, on the other, simulation, modeling, and computational technologies, provide invaluable support to interpret and integrate “omic” information and drive novel synthetic biology and personalized OV engineering approaches for effective immunovirotherapy. Altogether, these tools, possibly aided in the future by artificial intelligence as well, will allow for the blending of the information into OV recombinants able to achieve tumor clearance in a patient-tailored way. Various endeavors to the envisioned “synthesis” of turning OVs into personalized theranostic agents are presented.

## 1. Introduction

Biologicals based on oncolytic viruses (OVs) have been the focus of research and development for many decades now, with significant advances in providing a new line of treatment for particularly stubborn malignancies [1,2,3,4,5]. OVs are viruses able to specifically infect and replicate in tumor cells and are ultimately capable of destroying them without injuring normal healthy cells [6]. The immunogenic cell death (ICD) that follows OV infection induces local and systemic activation of the immune system against the cancer mass and is decisive for tumor clearance. Accordingly, OV therapy has been renamed immunovirotherapy [7,8]. OVs are only rarely wild-type isolates. More often, they are mutated viruses, carrying spontaneous mutations that give the desired anti-tumor phenotype, or viruses selected by in vitro directed evolution, while new-generation OVs are modified by rational genetic engineering. On the one hand, many virus species and families, including adenovirus, herpesvirus, measles virus, parvovirus, reovirus, and vaccinia virus, have been investigated and exploited, aiming at the gold standard of safety and efficacy (Table 1). On the other hand, many types of cancers have been chosen as targets (Table 2; [9]), especially the tumors that do not respond to standard therapies or have the most dismal prognosis [10,11,12,13]. Of note, OVs have to face many challenges, including the ever-evolving tumor targets that are able to adapt and escape by mutation and the immune system of the host, which can be friend (in immunovirotherapy) or foe (immunologic barriers to the virus) [14,15]. The OVs that have been authorized and are now in the clinic are just the tip of the iceberg of a plethora of viruses that have been generated starting 20–30 years ago with the design methodologies and engineering technologies available at that time. A relevant example is talimogene laherparepvec (T-VEC), the first (and so far the only) oncolytic immunovirotherapy approved by the FDA and EMA [16,17]. It derives from precursor herpes simplex virus (HSV) recombinants dating back to the 1990s, progressively improved by many engineering interventions (see [15]). The characteristics of tumor selectivity and specificity for safety issues were implemented first; then, arming with immunomodulatory molecules was added as it became evident that this was an essential requirement for efficacy [18]. The version for human use of the recombinant virus was challenged in clinical trials for the treatment of advanced melanoma [5,19,20,21,22]. This whole process took decades.

The design and optimization of OVs today require a forward leap, as it is inconceivable to test all combinations of possible cancers and candidate OVs. The intrinsic inefficiency, high costs, and huge time required by incremental improvements in candidates must ideally be substituted with methodologies and technologies able to forecast the most promising strategy and match between a cancer and an OV. Moreover, despite decades of efforts, OV immunovirotherapy proved to be effective in the clinic only against specific tumor types or in individual patients, while in many cases, it has shown no or very limited efficacy and does not add to standard therapies. These therapeutic failures arise from innate (primary), adaptive, and acquired resistance mechanisms that depend on cancer cell features, tumor heterogeneity, TME, immune system, and other host factors, as well as the fact that host and virus show multi-way and variable interaction [23,24]. Testing and scoring for specific tumor biomarkers is often the standard in assessing the suitability of a patient for a given therapeutic approach. However, the introduction of omic approaches can indeed help to capture the global blueprint of the tumor before and during treatment, making it possible to understand multiple aspects of the patient’s specific disease, propose a highly tailored therapy, assess the outcome, and rapidly introduce possible corrections to the therapeutic approach [25,26]. Today, fast-paced advances in genomics, big data analysis, synthetic biology, and artificial intelligence cast new light on the future design of oncolytic viruses by combining virus, tumor, and patient characteristics. Such an organic and wide-ranging approach will be the path for the implementation and prioritization of the more successful OV combination therapy. Here, we review the different approaches and efforts put in place to tackle the modeling and simplification of the complex settings of OVs in the host and tumor and have a glance at the opportunities of OVs as theranostic agents. We deal with OV vs. tumor mathematical modeling (Section 2); we confront systems and synthetic biology in OV design (Section 3); we analyze the opportunities for OV improvements offered by combination therapies and immune checkpoint blockade (ICB) (Section 4); we discuss the role of genomics, proteomics, and computational tools in OV design (Section 5); we highlight the advantages of large-scale assays (Section 6); and finally, we conclude with the perspective of theranostic OVs (Section 7).

## 2. Mathematical and Computational Models

Tumor cells and oncolytic viruses do not display constant interactions due to the complex interplay between cell and virus populations and the effects of the immune system. The frontline approach of testing viruses in homogeneous monolayer cell cultures shows its limitations by switching to 3D spheroid cultures. It must be considered that more complex simplifications like organoid cultures and, further, preclinical animal testing, only in part recapitulate what will happen in the clinical setting. Mathematical modeling comes into play to resolve the issue of first understanding and then predicting the impact of spatial dimension on the dynamics and efficiency of virus spread and, finally, of virotherapy. Berg et al. [27] analyzed the facets of this complex issue and informed a mathematical and computational model of virus spread by combining the results of in vitro 2D tumor cell cultures and 3D tumor spheroids, calculating the number of nearest neighbor tumor cells, and correlating the outcome of virotherapy with such dimensional and numerical parameters. The paradox was highlighted that a 3D environment has a higher number of neighbor cells and reaches equilibrium in a shorter time, but tumor eradication is more difficult to achieve than in a 2D setting. They underscore that other methods, like the non-spatial mean field computational models that do not explicitly include the parameter of space, usually provide an overly optimistic view of the success of virotherapy by underestimating the blockage imposed by the 3D spread. This is apparent experimentally, as in tumor spheroids, many infected cells may not be killed by oncolytic measles virus (oMV) infection, and many cells are not infected even if they are adjacent to infected cells [27]. A further aspect of spatial dynamics of oncolytic virotherapy was analyzed by Bhatt et al. [28], namely the effect of virus-resistant cells in the tumor mass, either through the presence or generation of tumor cells refractory to viral infection or through the occurrence of healthy (non-tumor) stromal cells, which are mostly naturally resistant to OVs. These virus-resistant cells act as spatial barriers to the spread of OV infection and contribute strongly to the therapeutic failure of oncolytic virotherapy. Since they are a property of any tumor, resistant cells must be considered when evaluating the possible outcome. The authors designed a computational model that could include the role of all three players (virus, tumor, and stromal cells) with a special focus on virus-resistant tumor cells and with the assumption that the advantage of being resistant to OV infection may impose a physiologic and metabolic cost to these cancer cells that affects their proliferation [28]. Thus, many parameters related to cells and virus, and combinations thereof, were used for repeated simulations, including the time of treatment with the virus, the stage and size of the tumor, the probability of the generation of resistant cells, the initial presence of stromal cells, the rate and route of virus spread (cell-to-cell or to distant cells), and the rate of division and death of cells (either infected or non-infected). Interestingly, repeated runs with neighboring (but also constant) sets of parameters could lead to a different outcome among four possibilities (total tumor eradication, partial tumor eradication, sensitive cancer persistence, and resistant cancer persistence) in a stochastic, non-deterministic way. This model recapitulates and may explain the discrepancy in virotherapy outcomes in clinical settings and why therapeutic failure occurs. The value of these models is that they allow us to partially unravel the intricate processes of oncolytic virotherapy and resistance by providing an understanding of their dynamics in a spatial framework. Other approaches have been proposed to dissect oncolytic virotherapy in interaction with the tumor microenvironment (TME) and, in particular, with immune system players, e.g., macrophages. They rely on ordinary differential equation (ODE) models that are especially useful for dynamical systems and systems biology and employ a combined analytical–numerical approach [29]. This method allowed to simulate and calculate the relative effect of various parameters, like virus spread, virus yield, and macrophage polarization/re-polarization rates, on the efficacy of virotherapy. A mathematical approach was also used to investigate macrophage plasticity in interaction with T cells and the OV to interpret the diverse kinds of immune responses that can lead to immunological tumor control and elimination [30]. The caveat is that the complex parametrization of the model depends on the availability of data to allow quantitative predictions. Other theoretical approaches included the simulation of the geometry of OV injection into a Voronoi cell lattice-based model [31], observing that efficacy is enhanced by repeated off-center injections and that delaying virus infection allows for prolonged and better virus dissemination and, eventually, a better therapeutic outcome. The implementation of further mathematical models holds promise to better inform especially the scheduling and timing of oncolytic virotherapy according to the virus and the tumor features.

## 3. Systems and Synthetic Biology for OV Therapies

(Multi)-omics big data analysis and their integration via systems biology come into play to determine the networks and patterns of gene expression and miRNA levels, protein interactions and processing, biochemical interconnections, and responses of cell populations (tumor and immune system) in the tumor specimens obtained from the clinic. Using this knowledge, systems biology can compute tumor features, intrinsic and acquirable resistances, vulnerabilities, and the behavior of the host immune system, and can determine whether specific OVs—or other approaches—are suitable for the patient or have to be excluded a priori for probable ineffectiveness (OV prioritization in clinic), and can define prognostic marker panels to monitor the evolution of cancer features during the therapy. This approach has been successfully applied to test a panel of RNA-OVs against glioblastoma (GBM) [32], a heterogeneous cancer type that rapidly develops resistance to therapies and where often OV treatment, assayed in clinical trials, does not extend life expectancy and does not recapitulate the results obtained in preclinical trials (see [24]). The multi-omics analysis of patient-derived GBM cultures correlated the sensitivity of the cells to be infected by RNA-OVs to transcriptomic and proteomic portraits typical of activated interferon (IFN) pathways, indicating a preserved IFN response in OV-resistant cells, while the cells susceptible to infection were defective in IFN pathways. Although this correlation was not surprising, the high occurrence of tumors or their subpopulations with preserved IFN response was unexpected, and the authors correlated this incidence to the high rates of OV failure in GBM. Determinants of resistance to OVs were pinpointed by omics and single-target assays in other types of cancers and for different OV families [33,34,35]. In addition to IFN pathways, factors for resistance to OVs included expression levels of virus attachment and entry receptors, availability of nucleotide pools, secretion of fibroblast growth factors, activation of mTOR and/or NFκB, modulation of apoptosis and stress response, and other factors [36,37,38,39]. It is worth noting that some of these factors have broad antiviral functions, while others are specific for OV classes (e.g., RNA/DNA viruses), OV families, or single OVs. Representative examples are the OVs derived from HSV (oHSVs): most of these OVs, including the FDA-approved T-VEC, are ablated for viral factors that counteract the IFN response so that they can replicate in IFN-defective cancer cells and not in IFN-competent healthy cells. In other oHSVs, cancer tropism is obtained by inserting cancer-specific heterologous regulating elements (miRNA responsive elements—MRE—or promoters/operators) upstream essential or virulence genes to allow virus replication only in the cancer cells that do not express the corresponding repressors or that express the necessary transcription factor [40,41]. In different approaches, tumor specificity is based on receptor retargeting [15,42,43]. Each modification to convert HSV into an OV requires that the target cancer cell harbors the corresponding feature. Tumors or subpopulations thereof that are or become negative for these characteristics negate oHSV functionality. Hence, the tumor blueprints produced by omic big data analysis and systems biology are fundamental for OV prioritization and the discovery of resistances.

Many technologies and omic approaches are involved in building maps in tumor and non-tumor cells, including transcriptomic and single-cell RNA sequencing, RNAi screenings, Cas9-guided mutagenesis [44], proteomic and phosphoproteomic [45,46], tumor immunopeptidomic [47], determination of T-cell receptor (TCR) clonality and targeting, radiomics [48], organ-on-chip and tumor-on-chip bioimaging [49], and others. Detailed repositories of tumor and cancer cell lines are now available for consultation [50].

OVs are also armed to increase the potency of the therapy. Cytokines, e.g., IL-12 [51,52,53] or GM-CSF [54,55,56], chemokines [53], or antagonist molecules like vasculostatin [57], have been engineered in OVs and proven useful to enhance efficacy in preclinical studies by activating the immune system, improving immune cell trafficking, or reducing the tumor invasiveness. The elicited molecular pathways are very different depending on the arming molecules and tumor specificity, so there is no universal arming strategy. Alternatively, the disarming strategy or attenuation described above, which can be extended to other viral factors involved in neutralizing the host’s antiviral systems, can cause an increased inflammation of the tumor and its TME that can override the immunosuppressive state and skew into an antitumor response [58]. Notable examples are ICP47 gene deletion in some oHSVs [59] and E3/19K in adenovirus. These factors normally limit viral antigen presentation, and their ablation increases the immunogenicity of infected cells. Similarly, factors that counteract IFN secretion by infected cells can be deleted or conditionally expressed to increase IFN levels in the TME and enhance tumor inflammation [41,60,61], along with determining cancer-tropism and the safety of these OVs.

Among all these possibilities, the future stands in the ability to engineer OVs with genetic circuits able to sense tumor cells or the TME state, activate different pathways, and tune the more appropriate gene expression for tumor eradication [24]. This would be particularly useful to treat metastatic tumors that may acquire different characteristics in different metastatic body districts [62] or to tackle the development of resistance to OVs. To design such improvements, a great input comes from synthetic biology for the design of genetic circuits that regulate the localization, timing, and extent of the expression of therapeutic payloads (or even viral genes for OV replication), along with the guidelines from systems biology. Synthetic regulatory circuits allow finer regulation and restrict OV payload expression only in target cells with the appropriate molecular features, reducing off-target effects and increasing therapeutic efficacy and specificity. Important issues in the optimization of synthetic biology circuits are the size of the construct, which must fit the size of the carrier OV, the reduction in off-target effects, and the limitation of interference and interaction with gene expression of the infected cells. These latter issues can be overcome by using specific activators with their own target sequences, with the drawback (or advantage) that any heterologous gene product increases the immunogenicity of the infected cells. This approach allows us to achieve a high level of expression in the cell, avoiding dampening or unspecific activation by the target cell background gene expression (transcriptional noise) [63,64]. Systems biology can contribute to this field by providing the best candidates to be included in the rational design of payloads and synthetic gene circuits with the least complexity and maximal specificity to be implemented in programmable OVs against a desired target tumor [64].

Recently, the engineering of an oncolytic Adenovirus (oAd) was described with a genetic circuit module made of a tumor-selective promoter and two reciprocally inhibitory heterologous repressors under the control of a pair of tumor- or healthy cell-specific miRNAs [65]. Thus, using this circuit, the effector molecule expression was restricted to cancer cells by multiple controls. Such a sophisticated genetic circuit was compacted into a small cassette of only 6.5 kbp and assayed with reporter genes and effector molecules like IL-1, GM-CSF, anti-PD-1 scFv, and anti-PD-L1 scFv to enhance the immunotherapeutic effect of oAd. The authors propose their circuit as a modulable genetic element that could be tuned for insertion in other OVs and for different tumors using specific promoter elements and pairs of miRNA/MRE [65]. Other authors developed synthetic circuits in oncolytic vaccinia virus (oVV) made with heterologous activators modulable by specific exogen compounds to control the expression of a reporter gene. Although this was a proof-of-principle, the circuits can be employed for controlled expression of payloads or viral genes [66].

## 4. Next-Generation Combination Therapies to Improve OV Efficacy

The analyses and predictions described above must be extended from the tumor to the tumor microenvironment, as well. The molecular pattern of cancer cells and TME has been shown to play a role in the resistance to anti-cancer therapy, including those based on OVs [67]. Indeed, the TME includes stromal cells, anomalous vasculature, and extracellular matrix that hinder access of OV to tumor cells. Moreover, the TME synthesizes an array of ligands, cytokines, and chemokines able to dampen the cytotoxic T cell functions and twist the immune cells into an immunosuppressive phenotype or to exclude them from tumors, achieving immunodesert cancer masses [68]. Cancer immunotherapy with drugs for T-cell immune-checkpoint blockade (ICB) has demonstrated high efficacy and improved life expectancy for some types of tumors, although the relatively low overall response rate and toxicity after systemic administration mar the therapeutic approach [69]. Accordingly, scoring positive for the immunological molecular targets is a prerequisite for ICB. Many preclinical and clinical studies showed the ability of OVs to prime tumors for ICB therapy by increasing their immunogenicity and infiltration by anticancer effectors [70,71]. In addition, OVs can be engineered to express molecules for T-cell ICB, inhibitors of the macrophage “Don’t Eat Me” signals, or immune-activating receptors (cytokines, chemokines, 4-1BBL, OX40L, CD70, GITRL, CD40L, etc.) from the cancer cells, granting high levels of these factors within the tumor bed without toxicities and severe side effects of a systemic administration [8,72,73]. As mentioned, blueprinting tumor and TME using omic approaches before and during OV treatment can identify markers for prioritizing OV arming and/or combination with ICB. The exceptional successes of OV-ICB combinations observed in preclinical studies were not recapitulated in clinical trials, as only a few proved superior efficacy with respect to ICB monotherapy. This indicates the need for deeper knowledge of tumors, TME, and host immune system in clinical settings to optimize schedule and timing to induce immune activation by ICB against the tumor and not against the OV [8,74]. Big omic data analysis, systems biology, and modeling can help in the optimization of combination therapy.

OVs can also be administered in combination with compounds that enhance viral replication. JAK-STAT inhibitors target the pathways downstream of IFN activation and allow OV replication in cancer cells with partially intact IFN signaling [75]. Similarly, NF-κB blockade increased OV replication and therapeutic efficacy [76]. Cyclophosphamide (CPA), which is used against several types of cancer, has been employed at high doses to temporarily inactivate the immune system and allow OVs (HSV, Ad, MV, reovirus, and VACV) to infect and replicate within the tumor at much higher levels, resulting in increased oncolysis, higher release of PAMPs and DAMPs, and ultimately increased anticancer immune response after recovery from CPA treatment [77,78]. Finally, some anticancer compounds such as CDK4/6 inhibitors that interfere with the cell cycle have been successfully combined with oAd therapy, as the virus benefits greatly from the induced modifications in terms of replication and persistence and shows higher oncolysis and immune activation [79,80,81]. OVs have also been combined with chemotherapeutics to increase the immunogenicity of dying tumor cells. Specifically, chemotherapies known to induce low levels of ICD were found to be improved by co-administration of OVs, as the latter increased cytotoxicity and ICD of cancer cells and resulted in better activation of the immune system against the tumor [82].

A high degree of complexity has been reached with oncolytic Adenovirus, merging arming and delivery strategy to synergize with and improve the efficiency of the CAR-T approach in a lung cancer model [83]: the ability of oAd to disrupt the TME was used to prime the tumor milieu for the best CAR-T performance. The difficulty of oAd to reach and penetrate solid tumors was circumvented by using mesenchymal stromal cells (MSCs) as carriers. Thus, MSCs were loaded with a combinatorial binary Ad vector made of an oAd for cancer cell lysis and TME disruption plus a helper-dependent non-lytic Ad vector (HDAd) expressing IL-12 and a PD-L1 blocking antibody for sustained expression of the immunomodulatory molecules for CAR-T cells stimulation. In other reports on solid tumor models (melanoma and glioma), loading OVs (vesicular stomatitis virus or reovirus) directly onto CAR-T cells in vitro improved therapy compared to single therapy with OV or CAR-T [84] by evoking viral-induced inflammation in an otherwise immunosuppressive TME. OVs expressing specific payloads, such as IL-12, IL-15, and IL-18, have also been employed to enhance the engagement and survival of endogenous NK cells in the anticancer response [8], as well as to empower adoptive CAR-NKs therapy against breast cancer brain metastases in preclinical models [85].

Another notable endeavor was to assay a triple combination therapy using the oHSV G47Δ platform. G47Δ (also Teserpaturev and Delytact) is an oHSV that was granted in 2021 conditional and time-limited approval in Japan for treating GBM and other malignant gliomas due to the encouraging results gained in phase 1 and 2 clinical trials on patients with progressive, residual, or recurrent glioblastoma [86,87]. G47Δ has also been assayed in pancreatic cancer, which is characterized by a particularly dense stroma, in combination with focal adhesion kinase (FAK) inhibition and ICB with anti-PD-L1 and anti-CTLA4 [88]. Remarkably, the triple therapy was able to remodel the tight tumor stroma, which normally supports tumor growth, allowing increased trafficking of immune effector CD8 T cells and CD4 T helper cells, and reduced infiltration by myeloid suppressor cells, overall enhancing the effects of the anti-tumor immunity prompted by the oHSV. The lack of efficacy of dual oHSV plus ICB therapy in these tumors with a supporting dense stroma makes it increasingly apparent that it is essential to act on TME structure and signaling, e.g., along the integrin axis, for successful immunovirotherapy [89].

The next frontier in OV combination therapy is the association with CRISPR-Cas gene editing technology, again exploiting the ability to use OVs as carriers of multiple biological systems, provided that the viral backbone has a suitable cloning capacity (see [90]). In this case, the OV delivers the gene editing circuit directly to the tumor or TME cells in a targeted way. Indeed, a few reports showed that this is feasible and adds efficacy to OV therapy alone. Pioneering work was performed on preclinical models of the pediatric cancer embryonal rhabdomyosarcoma (ERMS) with an oncolytic myxomavirus (family Poxviridae) engineered with a CRISPR-Cas9 targeting the signaling cascade of RAS, an undruggable oncogene. Following intratumoral OV administration, tumors were significantly reduced, and overall survival increased [91]. The drawbacks were some off-target effects of Cas9 and the emergence of mutations at the guide RNA cut sites, which highlighted the need for careful selection of the sites least prone to mutate and the use of Cas proteins with enhanced fidelity. A recent report adds some improvements to this work, such as the choice of Cas12a for its higher fidelity with respect to Cas9. In a model of lung cancer, CRISPR-Cas12a was cloned as payload into an Adenovirus vector and delivered intratumorally to disrupt EGFR expression [92]. The virus performed genetic reprogramming of the target cells without off-target effects, induced apoptosis, and, in some instances, led to complete tumor regression. Importantly, the combined action of the gene editing system was not detrimental to OV cancer-specific replication. Finally, the intrinsic immunogenicity of Cas proteins may be an added value for the enhancement of anti-tumor immunity.

## 5. Genomics, Proteomics, and Computational Tools for Oncolytic Immunovirotherapy

It is now accepted that the efficacy of OV therapies relies strongly on the contribution of the immune system in tumor clearance and the establishment of a long-lasting anti-tumor memory. Therefore, any new OV platform must confront the ability to prime and enhance systemic and local antitumor immunity while avoiding systemic side effects and utilizing strategies to overcome intrinsic tumor immunosuppression. One of the mechanisms and advantages of OVs is that during their replication in cancer cells, they can boost the exposure or release of tumor-associated antigens (TAAs) and tumor neo-antigens (TNAs) that may be otherwise elusive in the context of non-infected cancer cells. In addition, immune modulators expressed on the surface of immune cells or cancer cells may be targetable by OV-carried payloads. Similarly, the TAAs or TNAs more potent in stimulating a long-lasting response of cytotoxic T-cells could be cloned as payloads in OVs, with a pivotal role in immunotherapy success [93]. To date, many “omics” approaches are available to understand the multifaceted picture of possible ligands. Recent advances in immuno-peptidomics for the determination of the set of peptides presented on MHC-I [94] have led to the identification, in melanoma, chronic lymphocytic leukemia, and glioblastoma, of peptides originating from novel or unannotated open reading frames (nuORFs) as possible targets for immunotherapy [95]. Indeed, a large fraction of non-canonical antigens templated from sequences out of protein-coding regions or produced by non-canonical antigen processing (TNAs) both deriving from the dysregulation of cancer cells, as well as non-mutated proteins not expressed in healthy cells but abnormally expressed in cancer cells (TAAs), are believed to be specific or restricted to tumor cells. These antigens can be validated by the integration of advanced mass spectrometry results with transcriptomics, ribosome profiling, and bioinformatics tools [96]. Notably, the immunopeptidome undergoes changes in composition following OV treatment: a detailed analysis of the fluctuations may help to select the most appropriate candidates to be used as targets for personalized interventions [47,97]. A plethora of computational tools have been developed to cope with typical problems that may arise with oncolytic immunovirotherapy, including tumor resistance, sub-optimal viral spread, insufficient TME immunological heating, and the onset of anti-OV immunity. Such methods allow us to make predictions and rank TNAs, TAAs, anti-tumor peptides, and miRNA target sequences that can be engineered into OVs to obtain more effective and safer therapeutics [98]. Bioactive tumor-homing peptides (THPs) and anti-cancer peptides (ACPs) have many pros, including small size, ease of manufacturing and delivery to the target tissue, their specificity in tumor targeting, and the possibility of personalization and immunomodulation in vaccine formulations [99]. Their mechanisms of action include direct induction of apoptosis, autophagy, necrosis, or other cell death pathways. For these features, they are optimal payloads to be engineered into OVs to enhance the efficacy of virotherapy [100]. If engineered on the virus surface, they may also contribute to achieving or improving specific tumor retargeting of the OV. The design of ACPs [101] or THPs [102] can be aided by an array of computational tools, like AntiCP [103,104], ACPred [105], ACPred-Fuse [106] or ACPred-FL [107], MLACP [108,109], or TumorHPD [110,111]. Further TAAs and TNAs can be identified by analyzing the genomic profiles of cancer cells and screening for the best therapeutic index for their predicted ability to bind MHC molecules and elicit a CD4- or CD8-mediated antitumor immune response [112,113,114,115,116].

## 6. Large-Scale Assays for Oncolytic Immunovirotherapy

Several technologies are available to dissect the immunologic actors that are involved and the pathways that are elicited by and during OV therapy. These include genomic, transcriptomic, proteomic, and metabolomic analyses that can be carried out at the population or at the single-cell level [117]. All this information can be correlated with the outcome of therapy to identify factors and pathways, even those not apparently related to OVs and cancer, that may determine therapeutic successes and failures and used to select, improve, and combine OVs. In fact, data mining this huge amount of data allows the definition of markers for infiltration, polarization, and activity of different immune populations (cytotoxic T-cells, T-regs, DC, NK, B-cells, macrophages, neutrophils, etc.) involved in fighting or preserving different types of cancers to envision strategies to manipulate the activity of specific immune subpopulations and skew the immune responses elicited by OVs against the tumors [118,119,120,121,122]. Virus–host protein–protein interactions are organized in a number of molecular interaction databases that provide information per virus or per host [123,124] that can be browsed, searched, visualized, and downloaded for subsequent bioinformatic and network analyses. These databases can be the starting point for the OV-tumor interactome, for which only a few reports exist [125].

High-throughput, genome-wide RNA interference (RNAi) screenings directly test gene functions; therefore, they are potent tools to identify cellular non-oncogene pathways that could improve the efficacy of OV immunotherapy [126]. The identification of host factors expressed by immune or cancer cells that restrict infection by a specific OV allows the selection of the most appropriate backbone for OV-based therapy, as well as druggable targets to enhance oncolytic efficacy. A protocol for primary screening of oncolytic Maraba virus and Vaccinia virus has been implemented, and methods for secondary and tertiary steps of validation have been proposed [127]. Such methods can be easily extended to other OVs as well. RNAi screenings correlated ER stress to the sensitization of tumor cells to oncolytic rhabdoviruses [126], while reduced levels of ZfX or Mga proteins enhanced oncolytic Sindbis virus replication [128]. In the latter, it was also shown that shRNAs targeting virus-restricting factors can be engineered into OVs and specifically expressed in the infected cells to locally repress virus attenuation factors and enhance OV own replication. A large-scale screening of cytokines and chemokines linked high levels of betacellulin and IL-17F to resistance to many RNA-OVs [129]. Recently, a genome-wide CRISPR-Cas9 loss-of-function screening has been proposed to map the host factors responsible for the resistance of tumors to OVs by transducing single-guide RNAs (sgRNAs) into the genome of tumor cells [130]: the sgRNAs integrated in cells that survive OV treatment can be mapped by NGS sequencing and allow the identification of host factors involved in resistance to different OVs in multiple cancer models. Similarly, transcriptional analysis has proved that some tumors are able to acquire resistance to specific OVs by increasing IFN levels in the TME [131], and high-throughput screening of chemical libraries has been successful in identifying drugs that can neutralize these pathways and restore tumor sensitivity to OVs [132].

Omic technologies are also useful for identifying molecular signatures that determine the success or failure of anticancer therapies (chemotherapeutics, immunotherapy, and viral oncolysis) to be used as prognostic markers of the therapeutic approaches or to define druggable targets to improve clinical outcomes. Microarray analysis pinpointed low levels of immunoglobulin-like transcript 2 (ILT2) as a positive marker for the success of oVV-based therapy [133], while the failure of therapy with attenuated oHSV correlated to metabolic changes in the cells or variation of the viral receptors [38]. The study of molecular responses to OVs enables the identification of the best combination strategies against the tumor counteroffensive. In addition to the aforementioned IFN-dampening chemicals and CPA to enhance OV replication, large-scale approaches identified the most promising drugs for ICB for combination therapy [134], best candidates for OV-arming [135,136,137], and other factors required for increased OV replication [138], as well as the better cancer mutations for optimal OV-based therapy [139].

## 7. OVs as Theranostic Agents

Engineering with reporter genes presents numerous possibilities for OVs to be used as theranostic agents [140]. This has been proposed for phages, which are amenable to fast genetic engineering, synthetic assembly methods, chemical modifications, functionalizations, and refactoring to perform phage display [141,142,143,144]. Such modified particles hold promise to work for tumor imaging and therapy, and their ease of engineering allows them to cope with the standing evolution and adaptive behavior of tumors [141]. The plus of OVs with respect to phages is that they can replicate and amplify in target cells as both self-amplifying and self-limiting drugs and that they can express reporter genes upon reaching the target tumor and image the tumor while also exerting the anti-tumor activity [145,146]. This complex interaction between the diagnostic/drug (the OV) and the target (the tumor) again benefits from the integration of omic approaches and computational methods during both development and testing. As an example, the analysis of the secretome of tumors treated with OVs provides information on the mechanisms of immune activation induced by the OV itself and has a diagnostic value [147,148]. The optimization of integrated approaches for functional real-time imaging in clinical settings of OV replication, checkpoint expression, and immune cell trafficking is a priority for advancements in the field of OV immunovirotherapies [149]. To date, the most comprehensive OV theranostic reporter gene is the human sodium iodide symporter (hNIS), which enables the uptake of systemically administered radioisotopes by OV-hNIS-infected cancer cells. Accumulation of these species in cancer masses and metastases allows noninvasive nuclear medicine-based imaging of tumors via single photon emission-computed tomography (SPECT) and positron emission tomography (PET) and increases anti-tumor activity via internal irradiation (radiovirotherapy). OVs engineered for hNIS expression include oncolytic measles virus [150,151,152], Adenovirus [153], chimeric orthopoxvirus [154], vaccinia virus [155,156], and VSV [157]. The viruses engineered with hNIS were as safe and effective as the parental viruses. For an oncolytic Ad expressing hNIS, a phase I clinical trial was carried out, demonstrating that the treatment is safe and imaging is practical and valuable for monitoring viral replication and dissemination [158]. Similarly, an oncolytic MV-hNIS showed high therapeutic efficacy in preclinical models and phase I clinical trials against multiple types of cancers and allowed tumor imaging by ^123^I in a significant fraction of patients [159,160,161]. HSV thymidine kinase (HSV-TK) naturally encoded by HSV-based OVs or transgenically expressed by other OVs has been successfully employed in PET tumor imaging [162,163]. Other OVs were engineered with fluorescent, luminescent, and chromogenic reporter genes to visualize infected tumors. In animal models, these OVs were successfully employed for optical imaging of tumors and to evaluate in vivo virus biodistribution and specificity of infection (in-tumor and off-tumor). Although the very low transparency of human tissues to light limits the in-human translatability of the approach to superficial or accessible masses, the cancer-tropic GFP-expressing adenovirus OBP-401 has been successfully employed for the precise imaging of tumor margins during surgical resection (fluorescence-guided surgery) in mouse models of GBM, sarcomas, and metastatic carcinomas [164]. Finally, OV expressing melanogenic transgenes successfully enabled magnetic resonance imaging (MRI) and anticancer thermotherapy via self-generation of contrast and therapeutic agents [165].

## 8. Conclusions

**Figure 1:**
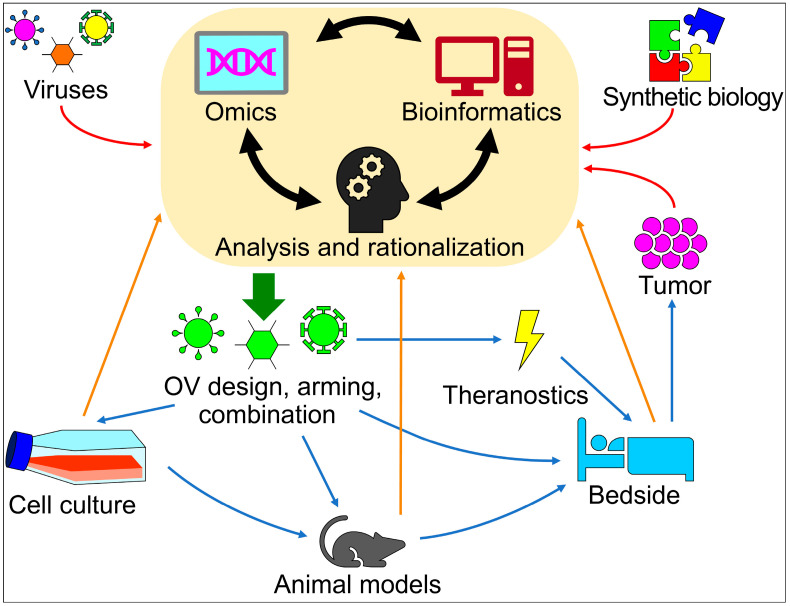
Schematic diagram of the integration of omics, bioinformatics, synthetic biology, and experimental, preclinical, and clinical data in the design of OVs, leading to the desired “synthesis” of engineering effective anti-tumor theranostics. Red and orange arrows indicate the flow of input information, and green and blue arrows the flow of output of data analysis and rationalization.

Tumor therapy is going beyond the low-throughput trial-and-error or incremental approaches typical of the pre-big data era. The improvements in power and reliability of the computational tools used to decipher and organize biological information are becoming of ever greater benefit in the field of OV design and oncolytic immunovirotherapy. OV’s special feature, even among biologicals, is the ability to replicate and self-limit in the patient and to have multiple interactions with different cell types, tissues, and organs besides the tumor itself and the TME. OVs are ideally designed and engineered to be targeted and armed against the tumor and to be safe towards healthy tissues and organs, avoiding off-target effects; shielding should be fine-tuned to avoid uptake by non-target clearance organs, like the liver and kidneys, or evade the attack of the immune system. These modifications must not hamper the ability to infect the target tumor, possibly via modified virus surface proteins. In the real world, OVs are attacked by the immune system but are concomitantly capable of instructing it against the tumor. In addition, OV biology may entail some variability or mutation rate according to their backbone (RNA vs. DNA), which must be taken into account in the perspective of specificity and safety. The picture is further complicated by the individual patient tumor characteristics. Omics integrated with synthetic biology and computational and mathematical models can offer the key to obtaining the urgently needed efficient and multi-tasking tailored biologicals able both to function in diagnosis and to be effective in therapy. The cartoon in Figure 1 illustrates this desirable scenario. The huge wealth of information coming from virus genomic sequences, synthetic gene circuits, and tumor molecular biology feed (red arrows) the “engine” of data analysis and rationalization that exploits omics approaches, computational models, and human intelligence. The output (green arrow) consists of prioritized engineered OVs that can be used alone or in combination in vitro, in vivo, and in humans (blue arrows). All these experimental or clinical settings produce new data that, in turn, again feed the data analysis engine (orange arrows) for improvements in OV immunotherapeutics or theranostics (yellow lightning symbol). OVs still have a hard time being approved by the authorities and getting into the clinic because there are almost no viruses that make it into Phase III trials. This underscores the need to start managing refractory and aggressive tumors via the factual merging and integration of human expertise with the inputs coming from huge dataset analyses, leading to the design and prioritization of the most promising engineered OV theranostics.

## Figures and Tables

**Table 1:** OVs employed in clinical trials ^1^.

Virus Backbone	OV Name	Number of Clinical Trials ^2^
Adenovirus (Ad, AdV)	DNX-2401; ONCOS-102; Enadenotucirev; OBP-301; Icovir-5; NSC-crAd; Ad-E6A7; Ad-MA3; Ad-MAGEA3; VCN-01; CG0070; CG7870; ONYX015; H101; TILT-123; LOAd703; ADV/HSV-tk; Ad5-yCD/mutTKSR39rep-ADP; DNX-2440; CAdVEC; NG-350A; ORCA-010; AdAPT-001; MEM-288; L-IFN; Ad-TD-nsIL12; ICVB-1042; NG-641	84
Coxsackievirus	V937	15
Herpes simplex virus 1 (HSV-1)	T-VEC; OrienX010; HSV1716; HF10; M032; G207; NV1020; rQNestin; C134; Rp1; Rp2; Rp3; ONCR-177; STI-1386; VG2025; T3011; R130; VG161; G47; Delytact	105
Herpes simplex virus 1 (HSV-2)	OH2; BS-006	16
Influenza	CodaLytic	1
Lymphocytic choriomeningitis virus (LCMV)	HB-302; HB-301	1
Maraba virus	MG1-E6A7; MG1-MA3; MG1-MAGEA3	4
Measles (MV)	MV-CEA; MV-NIS; MV-CD; MV-NAP	17
Newcastle disease virus (NDV)	NDV-HUJ; PV701; MTH-68H; MEDI5395; MEDI9253	6
Parvovirus	Parvoryx	2
Poliovirus-rhinovirus	PVSripo	11
Reovirus	Reolysin	36
Sendai virus	GEN0101	2
Seneca Valley virus	NTX-010; SVV-001	3
Vaccinia virus (VV)	Pexavec; GL-ONC1; JX-594; vvDD; TG6002; T601; TBio-6517; BT-001; OVV-01; RGV004; PF-07263689; VV-GMCSF-Lact; KM1; TG6050; hV01; CF33-CD19; JX-594; ACAM2000; MQ710; TG4023; CF33-hNIS; ASP9801	48
Vesicular stomatitis virus (VSV)	VSV-IFNβ; VSV-IFNβ-NIS; Revottack	9
Other (alphavirus; picornavirus; unspecified)	M1-c6v1; IVX037; RT-01	9

^1^ Query of ClinicalTrials.gov on 10 October 2023 with the keywords “oncolytic virus/viruses/virotherapy” and with OV families and names obtained by accessing the Pubmed database with the same keywords. ^2^ Some clinical trials employ multiple OVs.

**Table 2:** Cancer types targeted with oncolytic virotherapy in clinical trials ^1^.

Cancer Type	Number of Clinical Trials ^2^
Brain	43
Breast	33
Colorectal (CRC)	32
Gastrointestinal	16
Gynecological	32
Head and neck	29
Kidney	4
Liver	26
Lung	28
Mesothelioma	7
Neuroendocrine and peripheral nervous system	5
Pancreas	25
Prostate	8
Sarcoma	22
Skin	96
Urinary tract	21
Other solid tumors	43
Hematological tumors	18

^1^ Query of ClinicalTrials.gov on 10 October 2023 with the keywords “oncolytic virus/viruses/virotherapy” and with OV families and names obtained by accessing the Pubmed database with the same keywords. ^2^ Some clinical trials involve multiple cancer types.
